# Hybrid Interface in Sepiolite Rubber Nanocomposites: Role of Self-Assembled Nanostructure in Controlling Dissipative Phenomena

**DOI:** 10.3390/nano9040486

**Published:** 2019-03-27

**Authors:** Elkid Cobani, Irene Tagliaro, Marco Geppi, Luca Giannini, Philippe Leclère, Francesca Martini, Thai Cuong Nguyen, Roberto Lazzaroni, Roberto Scotti, Luciano Tadiello, Barbara Di Credico

**Affiliations:** 1 Department of Materials Science, INSTM, University of Milano-Bicocca, Via R. Cozzi, 55, 20125 Milano, Italy; e.cobani@campus.unimib.it (E.C.); i.tagliaro@campus.unimib.it (I.T.); roberto.scotti@unimib.it (R.S.); 2 Dipartimento di Chimica e Chimica Industriale, University of Pisa, Via Moruzzi 13, 56124 Pisa, Italy; marco.geppi@unipi.it (M.G.); francesca.martini@unipi.it (F.M.); 3 Pirelli Tyre SpA, 20126 Milano, Italy; luca.giannini@pirelli.com (L.G.); luciano.tadiello@pirelli.com (L.T.); 4 Service de Chimie des Matériaux Nouveaux, Centre d’Innovation et de Recherche en MAtériaux Polymères (CIRMAP), Université de Mons-UMONS, 7000 Mons, Belgium; Philippe.LECLERE@umons.ac.be (P.L.); cuong.nguyenthai@student.umons.ac.be (T.C.N.); roberto.lazzaroni@umons.ac.be (R.L.)

**Keywords:** sepiolite, nanocomposite, rubber, rolling resistance, filler reinforcement

## Abstract

Sepiolite (Sep)–styrene butadiene rubber (SBR) nanocomposites were prepared by using nano-sized sepiolite (NS-SepS9) fibers, obtained by applying a controlled surface acid treatment, also in the presence of a silane coupling agent (NS-SilSepS9). Sep/SBR nanocomposites were used as a model to study the influence of the modified sepiolite filler on the formation of immobilized rubber at the clay-rubber interface and the role of a self-assembled nanostructure in tuning the mechanical properties. A detailed investigation at the macro and nanoscale of such self-assembled structures was performed in terms of the organization and networking of Sep fibers in the rubber matrix, the nature of both the filler–filler and filler–rubber interactions, and the impact of these features on the reduced dissipative phenomena. An integrated multi-technique approach, based on dynamic measurements, nuclear magnetic resonance analysis, and morphological investigation, assessed that the macroscopic mechanical properties of clay nanocomposites can be remarkably enhanced by self-assembled filler structures, whose formation can be favored by manipulating the chemistry at the hybrid interfaces between the clay particles and the polymers.

## 1. Introduction

Over the last decades, research on nanocomposites (NCs) has stimulated enormous efforts in the development of improved functional properties [1,2,3]. Among NCs, the production of high performance polymeric NCs (PNCs) by incorporation in the rubber matrix of different nanoparticles, such as carbon black, carbon nanotubes, nanosilica, clays, layered silicates, and layered double hydroxides, strongly depends on their good dispersion in rubber [4,5], due to the interfacial nanofiller/polymer interactions and to the formation of a filler percolating network [6,7,8], which enable the material to support large dynamic loads over millions of load cycles [9,10]. 

On the other hand, the formation of a filler percolating network is considered to affect the total modulus together with the polymer network [6,7,8]. Indeed, the filler nanoparticles form interconnected structures, due to both direct particle interactions and their bridging by polymer chains, which enable the material to support large dynamic loads over millions of load cycles. Naturally occurring and readily available clays have been widely employed as environmentally friendly fillers for the production of high performance elastomeric NCs [5,11,12,13], owing to their structure and physical properties associated with two-dimensional confinement. However, the clay’s dispersion, distribution, and interfacial compatibility with the polymer still represent critical issues to be addressed in order to succeed in the successful development of high-quality clay-based PNCs. 

Until now, clay fillers embedded in rubber composites are generally modified by organic molecules (typically cationic surfactants) via an ion exchange reaction or by a grafting reaction with silane-based coupling agents. These surface treatments improve the dispersion of clays in the polymer as they favor the partial exfoliation of clay particles, forming tactoids of few layers (∼10) dispersed in the rubber through physical intermolecular interactions and mutually separated by thick rubber layers (∼10–50 nm), without the formation of any percolating filler framework [14]. In these conditions, the reinforcement is in charge of the hydrodynamic effect [15], depending on the shape factor and the filler volume fraction of the particles [16]. In fact, only at high loading organoclay fillers form particle aggregates, which substantially boost the modulus of the composite. However, the absence of stronger chemical bonds between clay and rubber chains precludes their homogeneous distribution and organization in the matrix in an effective filler network, causing a severe increase of the dissipative effects [17]. This has important technical implications in many elastomer applications involving dynamic loading as tire materials, especially for the reduction of rolling resistance (the mechanical energy converted into heat by tire moving on the roadway) directly linked to fuel efficiency and emission reduction [18]. Thus, in order to provide clay-based rubber materials suitable for tire applications, the reinforcement effect should go hand in hand with the formation of an extended percolative network, a good filler dispersion and distribution, and an effective interaction of clay particles with rubber. 

**Scheme 1:**
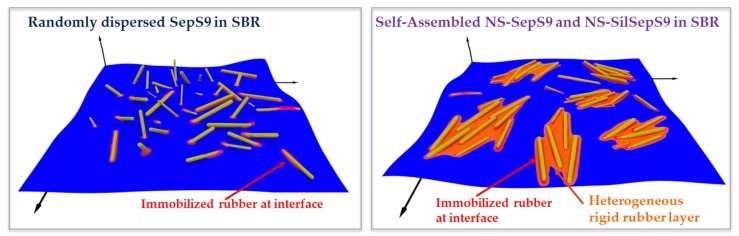
Schematic representation of self-assembly of modified sepiolite fibers (nano-sized sepiolite S9, namely NS-SepS9 and nano-sized silanized sepiolite S9, namely NS-SilSepS9) in styrene butadiene rubber (SBR) in comparison with randomly dispersed pristine sepiolite (SepS9).

In this frame, we succeeded in the preparation of PNCs based on nano-sized sepiolite (NS-SepS9) fibers, obtained by applying a controlled surface acid treatment, also in the presence of a silane coupling agent (NS-SilSepS9 fibers) [19]. The reduced particle size and the improved density of the surface silanol groups of Sep fibers allowed a better balance between the reinforcing and hysteretic properties of the rubber materials to be obtained, in comparison with the untreated bare Sep and silica particles conventionally used to reinforce rubber composites, leading to remarkable mechanical performances. These have been primarily related to the enhanced interfacial chemical interaction between modified Sep fibers and rubber, as well as to the size and self-assembly of anisotropic nanofibers in rigid filler network domains, as shown in Scheme 1. 

This work aims to investigate the influence of the Sep particles and self-assembled Sep particles on the dynamic-mechanical behavior of the styrene butadiene rubber (SBR)NCs, mainly focusing on the formation of the nanoscale rigid rubber at the Sep/SBR interface. With this in mind, herein, we report a detailed investigation at the macro and nanoscale of such self-assembled structures in terms of: (i) organization and networking of the Sep filler in the rubber matrix; (ii) filler–rubber interactions and; (iii) impact of these features on the reduced dissipative phenomena. Then, dynamic mechanical thermal analysis (DMTA) was utilized to assess the fraction of rubber with reduced mobility, which affects the stress/strain behavior of cured Sep/SBR PNCs. Low field ^1^H nuclear magnetic resonance (NMR) measurements were performed in order to determine the mobility of rubber chains, and, in particular, to highlight the changes in the mobility induced by the presence of the filler particles and by the vulcanization process [20,21,22]. The morphology of the PNCs and the topography of the immobilized rubber were investigated by the integrated approach of transmission electron microscopy (TEM) with atomic force microscopy (AFM) to visualize the nanomechanical properties in the elastomer zone at the interface between the matrix and the Sep nanostructures.

## 2. Materials and Methods 

### 2.1. Materials 

Sep Acid treatment: Sep Pangel S9 (SepS9) was purchased from Tolsa (Madrid, Spain); Bis(3-triethoxysilylpropyl) tetrasulfide (TESPT), ammonium hydroxide, isopropanol, and 37% aqueous hydrocloric acid (Sigma-Aldrich, St. Louis, MO, USA) were used without any further purification. Milli-Q water with a resistivity of 18.2 MΩ cm was used. 

Preparation of Sep/SBR PNCs: SBR (SLR 4630 from Styron Europe GmbH) had 25% styrene, 63% vinyl, and 12% butadiene; Treated Distillate Aromatic Extract (TDAE) extender oil (37.5 parts per hundred rubber (phr)) was obtained from Sigma-Aldrich (St. Louis, MO, USA); antioxidant *N*-(1,3-dimethylbutyl)-*N*′-phenyl-p-phenylendiamine (6PPD), Santoflex-6PPD, was supplied from Flexsys (Solutia Inc., Saint Louis, MO, USA); the curing agents were purchased as follows: Stearic acid (Stearina TP8) from Undesa (Barcelona, Spain); sulfur from Zolfoindustria (San Cipriano Po, PV, Italy); zinc oxide (wurtzite, specific surface area 5 m^2^ g^−1^) from Zincol Ossidi (Bellusco, MI, Italy;); *N*-cyclohexyl-2-benzothiazole sulfenamide (CBS) Vulkacit CZ/C from Lanxess (Cologne, Germany).

### 2.2. Preparation of NS-SepS9 by Acid Treatment

NS-SepS9 sample was prepared according to the previously published procedure [19]. 120 g of pristine SepS9 were dispersed in 1.2 L of isopropanol and stirred at 65 °C for 30 min. After adding 480 mL of 37% aqueous HCl solution, the mixture was mixed at 65 °C (600 revolution per minute (rpm)).

After 2 h, the NS-SepS9 fibers were collected by centrifugation and the powders were washed several times with deionized water and aqueous ammonium hydroxide (60%) in order to remove chloride anions, until pH 7 ± 0.2. Finally, the obtained solid was dried in an oven at 120 °C for 48 h.

### 2.3. Preparation of NS-SilSepS9 by Acid Treatment and Silanization

As previously reported [19], the acid treatment of pristine SepS9, performed as reported before, in the presence of TESPT silane (64.7 g), produces the funzionalized Sep, namely NS-SilSepS9. 

### 2.4. Preparation of Sep/SBR PNCs

In order to prepare uncured PCNs, Sep fillers were mixed by ex situ blending with SBR in a Brabender Plasti-Corder^®^ Lab-Station (Brabender GmbH & Co. KG, Duisburg, Germany), with a mixing chamber of 65 mL and filling factor of 0.6. In detail, SBR polymer was plasticized into the mixer for 30 sec at 60 rpm at 145 °C. Then, the filler (pristine SepS9 or modified NS-SepS9 or NS-SilSepS9, 35 phr) and TESPT (2.8 phr) were introduced and mixed for 2 min. Vulcanization compounds were added in two different steps. Firstly, 6PPD (2 phr), zinc oxide (3.5 phr) and stearic acid (2 phr) were mixed with the composites at 60 rpm for 5 min. at 145 °C. Successively, CBS (3 phr) and sulfur (1 phr) were introduced and mixed at 60 rpm at 90 °C. Finally, the uncured composites were milled in a two-rolling mill at 50 °C for 3 min. to obtain sheets of a 0.3 cm thickness, suitable for the vulcanization. Herein, uncured samples are called SepS9/SBR, NS-SepS9/SBR, and NS-SilSepS9/SBR. 

Cured composites were obtained by the vulcanization performed using a hydraulic press at 170 °C and 100 bar for 20 min. and labelled V-SepS9/SBR, V-NS-SepS9/SBR, and V-NS-SilSepS9/SBR.

Reference materials, prepared without any filler and silane coupling agent, were called SBR-REF-0 and V-SBR-REF-0.

### 2.5. Dynamic Mechanical Thermal Analysis (DMTA)

A DMA Q800 apparatus (TA Instrument Inc, New Castle, DE, USA) was used to investigate the viscoelastic properties of the Sep/SBR composites. In detail, the storage modulus (E′), loss modulus (E″), and Tan Delta (Tan δ) of each composite sample were measured as a function of temperature, by applying a tensile stress mode (tensile strain of 0.1%). 

The dimensions of the rectangular specimens were a 3 mm width, 1.5 mm, and 15 mm length. Specimens were tested in the flexure (dual-cantilever bending) mode, by applying a preloaded force of 0.1 µN. All samples were heated at a constant rate of 3 °C min^−1^ from –40 to 80 °C and tested at a frequency of 1 Hz.

Two measurements were carried out for each sample. 

### 2.6. Solid State NMR Analysis

Low-resolution solid-state (SS) NMR experiments were carried out on a spectrometer made of a Stelar PC-NMR console (Stelar s.r.l., Mede, PV, Italy) interfaced with a Niumag permanent magnet, working at the 1 H Larmor frequency of 20.8 MHz. The temperature was always in the range of 24 ± 0.1 °C. On-resonance ^1^H Free Induction Decays (FID’s) were acquired using a Solid Echo (SE) pulse sequence, in order to also detect fast-decaying components characterized by short spin-spin relaxation times (T_2_). Quantitative data were obtained by acquiring solid echo FID’s at different echo delays from 13 to 31 μs, and extrapolating signal intensities to a zero delay. The T_2_ of the long-decaying components of the FID were measured by means of Carr-Purcell-Meiboom-Gill (CPMG) experiments with an echo delay of 13 μs. The 90° pulse duration was 3 μs. A relaxation delay of 1 s and 200 scans was always used. The experimental solid echo FID’s and CPMG relaxation curves were analyzed by a discrete approach using a non-linear least square fitting procedure implemented in the Mathematica^®^ environment (Wolfram Research Europe Ltd, Oxfordshire, United Kingdom).

### 2.7. Morphological Characterization of Sep/SBR PNCs

Preliminary morphological investigation was carried out on vulcanized composites by using a Zeiss EM 900 microscope (Zeiss, Oberkochen, Germany). Specimens (about 50 nm thick) were cut at –130 °C by using a Leica EM FCS cryo-ultra microtome (Leica Microsystems, Wetzlar, Germania). 

The microscopic morphology of the PNCs was also studied with scanning probe techniques. For that purpose, the samples were freeze-fractured in liquid nitrogen to reveal their internal structure and several complementary AFM-based techniques were employed, using a Dimension Icon (Bruker, Billerica, MA USA) microscope: Besides ‘classical’ Tapping-Mode AFM (TM-AFM), which mostly provides information on the morphology, Peak Force Tapping AFM (PFT) [23] and Intermodulation AFM™ (ImAFM) [24] were used to map the nanoscale mechanical properties.

Compared to TM-AFM, PFT uses much softer cantilevers (k < 1 Nm^−1^), allowing for an optimal combination of high force sensitivity and controlled surface deformation. In those conditions, the probe gently makes contact with the surface in each measurement cycle with a controlled force ranging from a few nN to a few pN (depending on the sample stiffness), which generates an indentation as modest as 1 to 2 nm. To extract quantitative information on the mechanical properties from PFT measurements, the cantilever is calibrated on a hard and clean surface (i.e., silicon) prior to the experiment on the sample under study.

In ImAFM, the cantilever is driven at two frequencies close to its resonance frequency. Upon interaction with the surface, frequency mixing takes place; this effect is called intermodulation [24] and it leads to a broad frequency spectrum, which is recorded with a lock-in amplifier [25]. The analysis of those intermodulation modes provides detailed information on the mechanical properties and viscoelastic response of the probed surface. 

In this study, RTESPA-300 (k ~ 40 N/m, Bruker, Billerica, MA USA) silicon cantilevers were used for both TMAFM and ImAFM measurements, whereas the PFT analyses were carried out with softer SNL probes (k ~ 0.12 N/m, Bruker, Billerica, MA USA). All images were recorded at a 0.5 Hz scanning rate, with a resolution of 512 × 512 pixels. 

## 3. Results and Discussion

### 3.1. Dynamo-Mechanical Analysis of Sep/SBR PNCs

DMTA was utilized to preliminarily assess the fraction of rubber with reduced mobility, which affects the stress/strain behavior of cured Sep/SBR PNCs. In detail, E′, E″ and Tan δ, in the temperature range from −40 to +80 °C) were monitored as a function of the different Sep fillers. 

**Figure 1:**
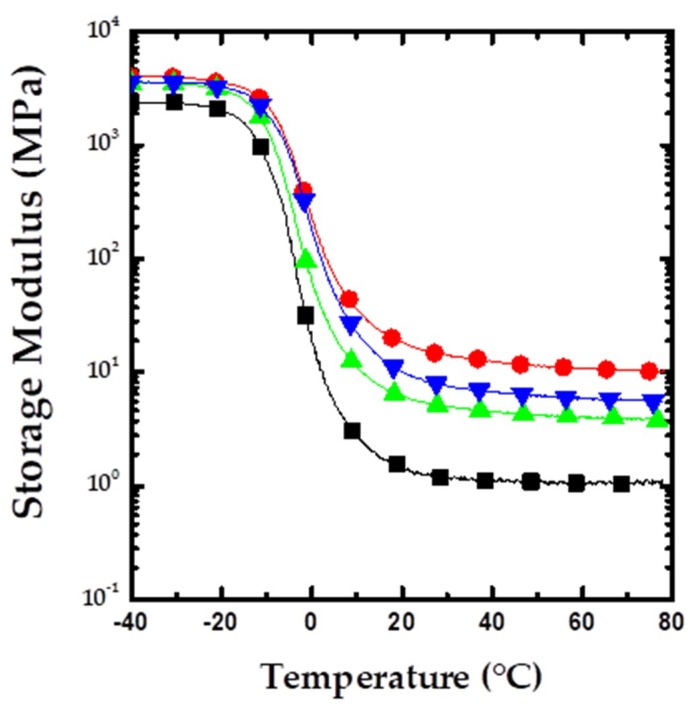
Storage modulus (E′) vs. temperature for a) reference material V-SBR-REF-0 (■—black line), vulcanized sepiolite S9/SBR nanocomposite (V-SepS9/SBR, ●—red line), vulcanized nano sized sepiolite S9/SBR nanocomposite (V-NS-SepS9/SBR, ▲—green line), and vulcanized nano sized and silanized sepiolite S9/SBR nanocomposite V-NS-SilSepS9/SBR (▼—blue line).

The evolution of E′ is plotted in Figure 1 for pristine and modified SepS9 in comparison with unfilled V-SBR-REF-0 composite. 

E′ reflects the elastic modulus of the rubber materials, which measures the recoverable strain energy in a deformed specimen. For all the samples, the sharp fall in E′ above −20 °C corresponds to the Tg region, as the modulus decreases drastically going from the glassy state toward the rubbery state. The modulus of the Sep/SBR composites is higher than that of neat SBR due to the reinforcing effect of the Sep fillers, which becomes more evident above Tg, T > 0 °C, when the rubber chains undergo fast segmental dynamics. Moreover, in the presence of the Sep fillers, the decreased region of E′ is shifted to higher temperatures. The reinforcement obtained for V-NS-SepS9/SBR (▲ line in Figure 1) and V-NS-SilSepS9/SBR (▼ line in Figure 1) is slightly lower than that of V-SepS9/SBR (● line in Figure 1) as expected for PNCs containing anisotropic particles with a lower aspect ratio (AR) at small strains in the rubbery state (constant strain amplitude of 0.1%). In fact, after acid treatment, the AR of Sep fibers decreases from 30 for pristine SepS9 to 4 and 23, respectively, for NS-SepS9 and NS-SilSepS9, as already reported [19]. The E′ value at 30 and 70 °C for the composites studied are listed in Table 1. 

**Figure 2:**
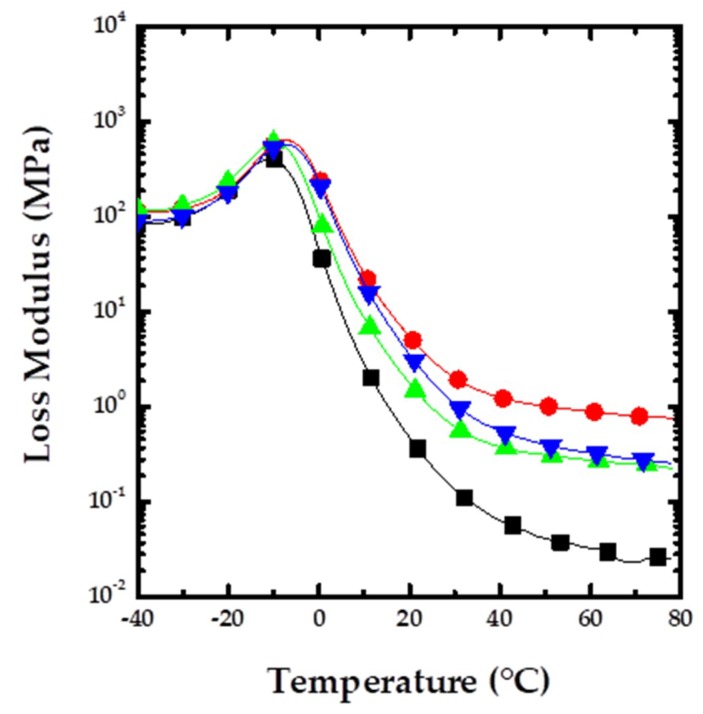
Loss modulus (E″) vs. temperature for V-SBR-REF-0 (■—black line), V-SepS9/SBR (●—red line), V-NS-SepS9/SBR (▲—green line), and V-NS-SilSepS9/SBR (▼—blue line).

Figure 2 shows the E″ variation for the different composite systems. Generally, E″ is a measure of the dissipated energy as heat per cycle under deformation, i.e., the viscous response of the material. The peak of E″ is at Tg, the temperature at which the material undergoes the maximum change in polymer mobility [25]. Above Tg, the E″ value decreases because of the free movement of the polymer chains. The Tg values of the Sep/SBR NPCs are reported in Table 1. The Tg values for V-NS-SepS9/SBR and V-NS-SilSepS9/SBR are slightly lower than that of V-SepS9/SBR related to the reinforcement effect due to their lower AR.

E″ increases over the entire temperature range upon the introduction of SepS9 (● line in Figure 2) in comparison with neat SBR (■ line in Figure 2). This may be attributed to the inhibition of the relaxation process within the composites as a consequence of a higher number of chain segments upon fiber addition. For the samples, V-SepS9/SBR and V-NS-SilSepS9/SBR, a slight shift in Tg toward higher temperatures (Table 1) can be observed, which can be attributed to the interaction of the SBR chains with the Sep filler [26].

It is worth noting that V-NS-SepS9/SBR (▲ line in Figure 2) and V-NS-SilSepS9/SBR (▼ line in Figure 2) show a significantly lower value of E″ in the rubbery region with respect to V-SepS9/SBR. This indicates that the modification of the Sep fibers introduces remarkable interactions with the rubber matrix, reducing the dissipative process. In particular, in V-NS-SilSepS9/SBR, the presence of the grafted silane allows a stronger filler–rubber interaction. The E″ values at 30 and 70 °C are listed in Table 1. 

**Figure 3:**
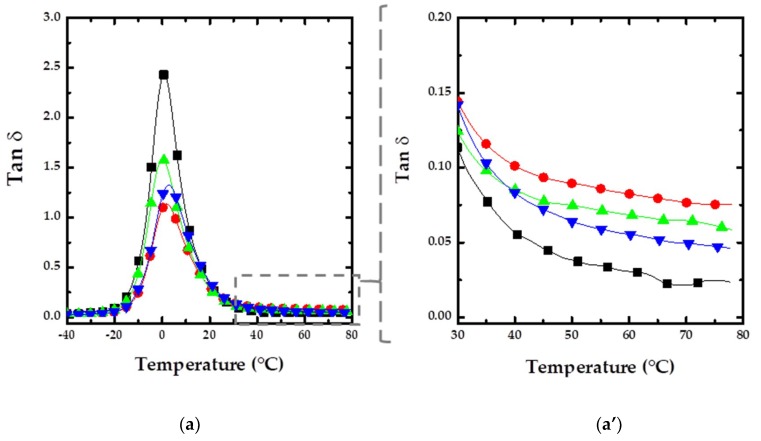
Tan δ vs. temperature of (**a**) V-SBR-REF-0 (■—black line), V-SepS9/SBR (●—red line), V-NS-SepS9/SBR (▲—green line), V-NS-SilSepS9/SBR (▼—blue line), and (**a’**) the corresponding magnification in the 40–80 °C range.

The effects of Sep fillers on damping, determined by Tan δ = E″/E′, are illustrated in Figure 3. In the composites, damping is influenced by the incorporation, type, and distribution of fibers, as well as by the fiber–matrix interaction and the void content. It is well recognized that Tan δ values are a good indicator of rubber material hysteresis and can be associated to the rolling resistance [27].

Interestingly, there is a significant effect of Sep fibers on the shape of the damping curve. In the presence of pristine (● line in Figure 3a) and modified SepS9 (▲ and ▼ lines in Figure 3a), the height of the damping maximum decreases while simultaneously the damping peak broadens. 

The compounds with Sep show a strong decrease in the maximum value of Tan δ compared to pure SBR. This can be ascribed to the stronger reinforcing tendency effect of the filler and to a reduced mobility of the rubber chains close to the filler or in the confinement of the filler network, due to physical and chemical adsorption of SBR chain segments on the particle surface. This reduces the amount of free rubber and consequently the magnitude of the loss factor peak. 

As an indication of skidding behavior (grip, traction) on dry roads, the level of the loss tangent in the range of 1–10 °C can be considered [27]. The range between 25 °C and approximately 70 °C comprises the running temperatures of a tire. Under these temperature conditions, the loss factor essentially determines the degree of rolling resistance. In particular, the Tan δ value at 70 °C can be considered as a direct probe of the rolling resistance. In our case, Tan δ values at 70 °C (Figure 3a’, Table 1) for both the modified SepS9 samples, especially for V-NS-SilSepS9/SBR, are lower than that of pristine Sep, demonstrating a reduction of the rolling resistance. This means that on increasing the temperature, nano Sep filled materials display a larger portion of the filler network, which can be broken down and reformed during cyclic deformation with respect to unmodified SepS9/SBR. 

The Payne effect, calculated as the difference between the modulus at low strain (G′_0_) and the modulus at high strain (G′_∞_), is in line with these observations. The Payne effect is widely accepted as a nonlinear trend of the modulus, as a consequence of the progressive destruction of the filler network under shear strain. Δ(G′_∞_–G′_0_) values are 0.152 for V-NS-SepS9/SBR and 0.077 MPa for V-NS-SilSepS9/SBR, significantly lower than that of the composite, V-SepS9/SBR (0.242 MPa), containing pristine SepS9 [19]. This suggests a strong immobilization of the polymer chains close to the nanofibers’ surfaces and within their network.

### 3.2. SS NMR Study of Sep/SBR PNCs

In order to investigate how the dynamics of the polymer matrix are affected by the presence of the Sep fillers, a low-resolution SS NMR study, based on measurements of 1H spin-spin relaxation times (T_2_), was carried out on both the unfilled SBR and Sep/SBR composites. A complete characterization of the dynamic properties was achieved by combining the SE and CPMG experiments [28]. SE was used to refocus the signal from the dipolar-coupled protons in rigid domains characterized by very short T_2_ (typically on the order of 10–20 μs), which would otherwise decay during the instrumental dead time. On the other hand, CPMG experiments were exploited to accurately measure the intrinsic T_2_ relaxation times of the long-decaying liquid-like components of the ^1^H FID, which can be artificially shortened by non-dynamic contributions, such as the magnetic susceptibility of the sample and/or fluctuations and inhomogeneity of the external magnetic field. 

**Figure 4:**
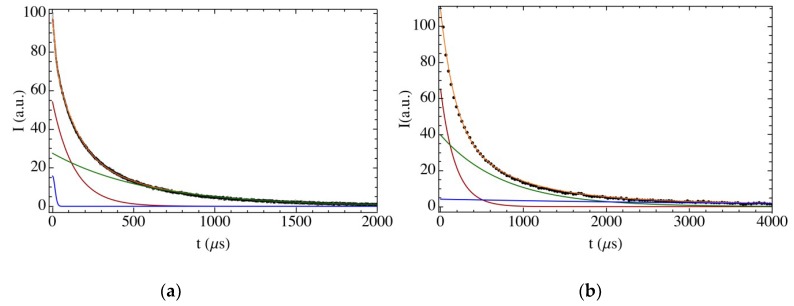
(**a**) Fitting of the on-resonance ^1^H Free Induction Decay (FID) (dotted line) recorded by Solid Echo (SE) pulse sequence for the sample V-NS-SilSepS9/SBR. The fitting function (orange line) and the single contributions of the Gaussian (blue line), short-T_2_ (red line), and long-T_2_ (green line) exponential components are shown. (**b**) Fitting of the Carr-Purcell-Meiboom-Gill (CPMG) relaxation curve (dotted line) recorded for the sample, V-NS-SilSepS9/SBR. The fitting function (orange line) and the single contributions of the three exponential components, exp1 (red line), exp2 (green line), and exp3 (blue line), are shown.

The discrete analysis of a ^1^H FID recorded on resonance by SE allows domains with different degrees of mobility to be identified and quantified. The ^1^H FID is fitted to a linear combination of functions (fi), each characterized by a spin-spin relaxation time, T_2i_, and a weight percentage, *wi*. The value of T_2i_, out of the so-called rigid lattice regime, monotonically increases with the increase of mobility, while *wi* is related to the percentage of protons belonging to the *i*-th domain [26,29,30]. Since, in the presence of multiple ^1^H–^1^H dipolar interactions, the efficiency of SE progressively decreases by increasing the echo delay (ED), quantitative values of *wi* can be determined by performing SE experiments at different echo delays and extrapolating the intensity of each component fit to a zero delay. For all the samples, the experimental ^1^H FID’s were well reproduced with a linear combination of two exponential and one Gaussian functions (as shown, as an example, in Figure 4 for sample V-NS-SilSepS9/SBR. The Gaussian function (Gau) is characterized by a very short T_2_ of ∼20 μs typical of protons located in rigid solid-like environments and could arise not only from rigid SBR domains, but also from protons located in Sep fibers. It is worth noticing that the rigid SBR domains can include bound immobilized rubber located at the Sep/SBR interface as well as entangled and crosslinked rubber chains [31,32]. 

On the other hand, the two exponential functions, representing from 84 to 95% of the ^1^H FID, are characterized by long liquid-like T_2_ values in the ranges of 150–250 and 500–800 μs, and are ascribable to protons in very mobile environments and/or to isolated hydroxyl groups of the filler. A more accurate description of the T_2_ relaxation behavior of these long-decaying components was obtained from the analysis of the CPMG relaxation curves. In all cases, the CPMG curves could be well fitted by a linear combination of three exponential functions, here indicated as exp1, exp2, and exp3, where T_2_ increases in going from exp1 to exp3. In particular, T_2_ values of ∼300, ∼1200, and ∼6500 μs and of 130–180, 580–760, and 3400–4500 μs were found for the uncured and vulcanized samples, respectively. An example of fitting is shown in Figure 4. These three exponential components provide a more complete and reliable picture of the non-rigid environments present in the samples. In particular, exp1 and exp2 indicate the presence of a distribution of mobile environments with different mobility, mainly ascribable to the SBR matrix, while exp3 can be assigned to SBR free chain ends or dangling chains. The decrease of the T_2_ values observed in passing from uncured to vulcanized samples agrees with a decrease of mobility of the rubber chains due to the formation of cross-links induced by vulcanization. In Table S1 of the Supplementary Materials (SM), the weights, *w_i_*, and T_2i_ values of the different protons fractions are reported. In particular, the reported values of *w_i_* were obtained by combining the results from the SE and CPMG experiments, assuming that the shortest- and longest-T_2_ exponential components of the ^1^H FID’s corresponded, respectively, to exp1 and to the sum of exp2 and exp3. The increase of the weight percentage of exp1 with respect to exp2 in passing from the uncured to the vulcanized samples further confirms the reduced mobility of SBR chains after vulcanization. For the uncured samples, neither the T_2_ values nor the weights of the three exponential components significantly vary in passing from pure SBR to the Sep/SBR (Figure 4), which are not directly involved in the interaction at the SBR/filler interface. On the other hand, in the case of the vulcanized samples, the T_2_ values are found to be slightly longer for the composites, V-SepS9/SBR and V-NS-SepS9/SBR, than for the unfilled SBR sample, V-SBR-REF-0, meaning that in the presence of filler, the SBR chains experience a higher degree of mobility. This result can be explained by the lower cross-link density of the rubber matrix possibly due to the inactivation of parts of the vulcanization system by adsorption onto the high-surface Sep filler. For V-NS-SilSepS9/SBR, shorter T_2_ values, more similar to the unfilled vulcanized sample, were found, possibly due to the formation of stronger SBR–filler interactions promoted by the surface silanization of Sep.

As already stated, for the SBR/Sep composites, the weight of the Gaussian component (*w* values in Table S1) accounts for all protons in rigid environments (f_Hrigid_^TOT^), from both rubber domains with restricted mobility and the filler. In order to investigate the influence of the different Sep fibers in the formation of a surface-immobilized rubber layer at the Sep/SBR interface, it was therefore necessary to separate these two contributions by determining the fraction of rigid protons belonging to the filler (f_Hrigid_^Sep^). To this aim the following strategy was used, similar to what done by some other authors [7,28]: (a) a calibration curve was built using weighted standard samples with known hydrogen contents, where the total ^1^H FID intensity to sample weight ratio was plotted against the hydrogen weight percentage (% wt/wt) (Table S2 and Figure S1 in the SM); (b) the total hydrogen content (% wt/wt) in each type of filler was determined by interpolating the calibration curve, after measuring the total ^1^H FID intensity of weighted pure Sep samples (Table S3 in the SM); (c) the ^1^H FID’s recorded for the pure Sep fillers were then analyzed in order to evaluate the fraction of protons in rigid environments (Table S2); (d) knowing the amount of filler in the composite samples, the fraction of protons from rigid rubber (f_Hrigid_^SBR^) was calculated as (f_Hrigid_^TOT^ − f_Hrigid_^Sep^). 

**Figure 5:**
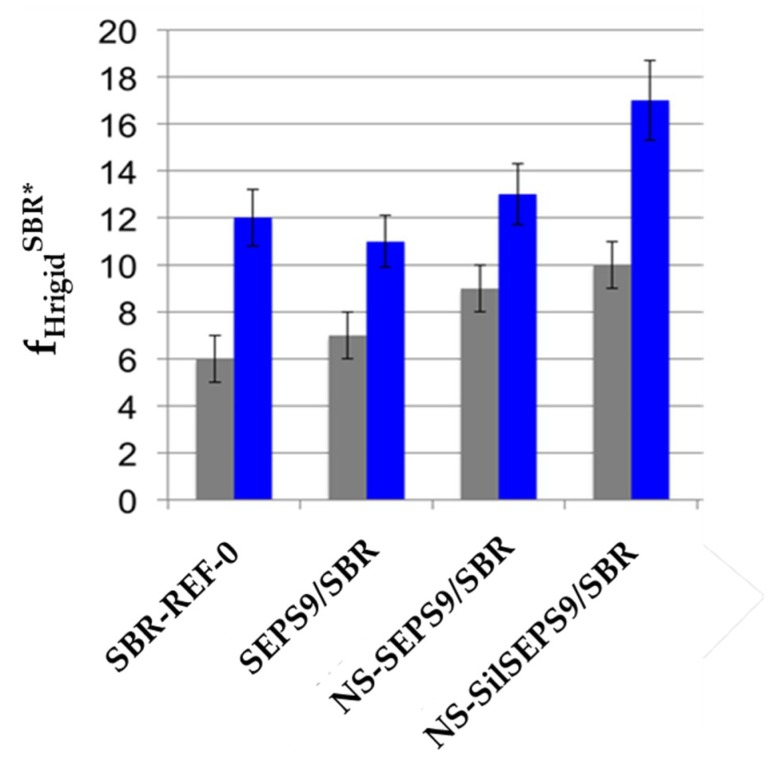
Fraction of rigid rubber (f_Hrigid_^SBR*^, %) determined for the uncured (grey) and vulcanized (blue) samples from the analysis of ^1^H FID’s, as described in the text.

The final results are reported in Table 2 and Figure 5. First, it can be noticed that the percentage of rigid SBR is significantly larger in vulcanized (11–17%) than in uncured samples (6–10%). This result was expected and could be explained by the higher amount of "immobilized" rubber chains after vulcanization. For the uncured samples, the presence of Sep fillers determined a slight increase of the amount of rigid rubber, in the order NS-SilSepS9/SBR > NS-SepS9/SBR > SepS9/SBR. Assuming that a possible contribution from the entangled rubber chains does not significantly change among the samples, this increase can be ascribed to the formation of immobilized rubber at the interface with the filler, which is enhanced when the modified NS-SepS9 and, especially, NS-SilSepS9 are used. 

With respect to the vulcanized samples, the NMR results indicate that surface-immobilized rubber layer forms, in particular in the presence of NS-SilSepS9 (Figure 5). Although the surface-immobilized rubber layer is only a small fraction, about 4% of the entire polymer matrix, it can affect the entire bulk and have a tremendous influence on the mechanical properties of the PNC material, since the immobilized rubber layer significantly alters the percolation and the morphology of the composites [32]. For example, the formation of strong interfacial interactions could be related to the strain independence of the storage modulus (Figure 1) and to the improvement of the hysteretic behavior observed for the PNCs with the modified Sep fillers (see Section 3.1).

Until now, it has been reported that clay particles, even if exfoliated and well dispersed, are not able to significantly interact with the rubber matrix. In fact, the mechanical properties in rubber-clay NCs, achieved without chemical bonds between the polymer and filler, have been mainly attributed to the geometric effect of stiff and well-dispersed filler particles. 

In order to calculate f_H_^Sep^, the presence of TDAE oil had to be taken into account, assuming an average hydrogen content of 10% (H%, wt/wt) of this oil. It was verified that by varying H% in the range of 9–11%, the calculated values of the different proton fractions remain within the experimental error. TDAE was added in the same amount to all the samples, and its contribution did not substantially affect the differences in terms of the amount of rigid rubber observed among the samples.

Therefore, the reinforcement mainly benefits from the increased modulus of the percolated mechanical network as compared to the soft matrix. On the contrary, the modification of the Sep fibers introduces a better interaction with the matrix, in particular for the NS-SilSepS9 sample and the formation of immobilized rubber, as demonstrated by NMR, thus reducing the dissipative process in the rubber NCs, as evidenced by the DMTA analysis (see Section 3.1). 

In addition, the self-assembly of nanosized Sep particles allows the organization of anisotropic fibers in filler superstructures where rubber chains are immobilized in the inter-particle region (see TEM and AFM analysis).

### 3.3. TEM and AFM Morphological and Nanomechanical Property Analysis of Sep/SBR PNCs

**Figure 6:**
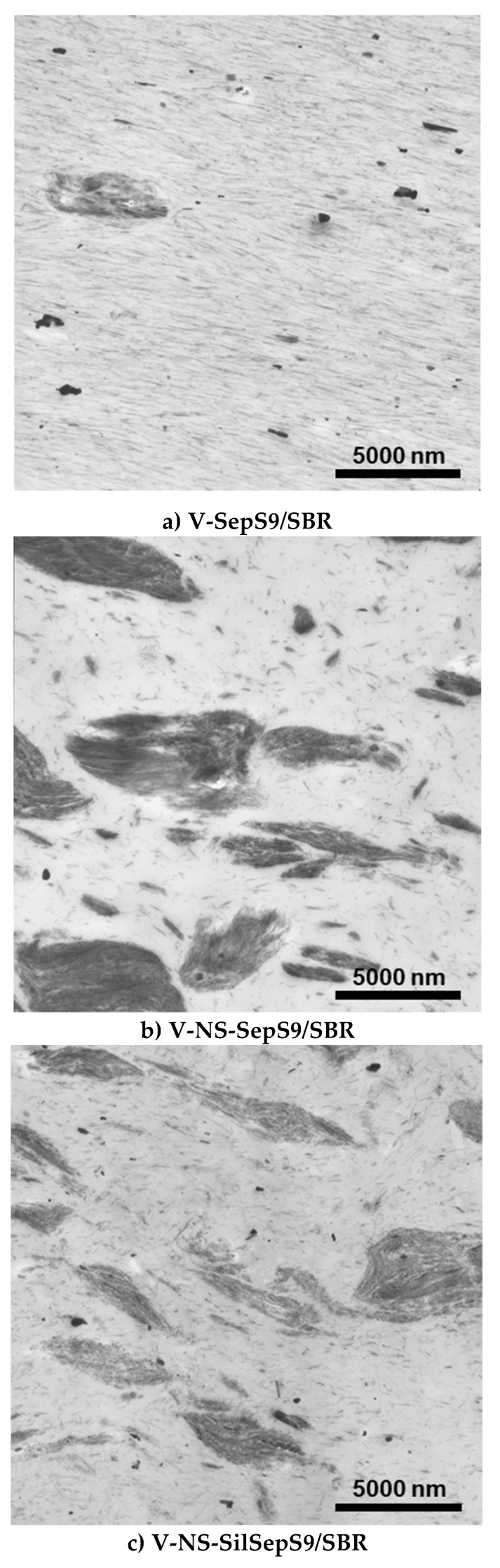
Transmission electron microscopy (TEM) images of (**a**) V-SepS9/SBR, (**b**) V-NS-SepS9/SBR, (**c**) V-NS-SilSepS9/SBR.

Although reported in a previous paper [19], some novel TEM images of V-NS-SepS9/SBR and V-NS-SilSepS9/SBR samples are reported, in order to more easily discuss the effects of the particle size and self-assembly on the rubber matrix (Figure 6). V-SepS9/SBR shows a uniform distribution and a continuous network of Sep particles (a in Figure 6). In V-NS-SepS9/SBR and V-NS-SilSepS9/SBR, after acid treatment, the AR of the particles decreases and a preferential alignment occurs along their main axis (b and c in Figure 6). This leads to the formation of domains of few aligned nanofibers whose orientation in the rubber matrix is different. Besides, TEM analysis of the composites shows that only the modified Sep fibers succeed in disrupting the tactoid interactions and favor the formation of a filler network. 

The filler self-assembly in these nanostructures provides larger amounts of overlapping rubber layers at the filler–rubber interface, as confirmed by the improvement of the mechanical properties.

Further evidence on the filler–rubber interactions occurring in the presence of surface modified Sep fibers were gained by investigating the PNCs morphology and the rubber/Sep fiber interface by AFM on fresh surfaces after freeze-fracturing the materials. 

In the context of these experiments, it should be taken into consideration that the preparation of these freeze fractured specimens could cause the cutting of fibers, lowering the probability that fiber particles completely lie on the fractured surface.

**Figure 7:**
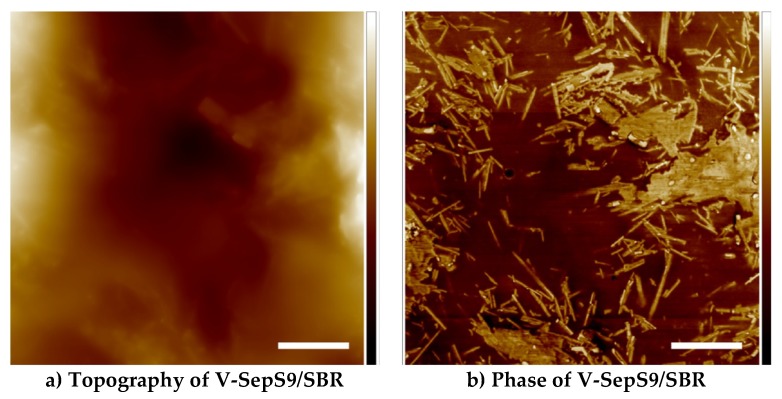
Tapping-Mode (TM) topographic (**a**) and phase (**b**) images of V-SepS9/SBR PNC. The white scale bar is 1.0 µm. The Z-range is 1.0 µm and the phase scale is 25 degrees.

The TM-AFM analysis was performed either with height images (a in Figure 7) or in phase contrast (b in Figure 7), which often reveals the domain morphology of thermoplastic [20] and other polymer materials. Phase images show a sharper contrast at the edges of the heterogeneous zones of the materials and give better evidence of the interface regions. 

Figure 7 shows the AFM images of the V-SepS9/SBR composite. It is evident that a remarkable contrast exists at the edges of the heterogeneous regions of the composite, corresponding to SepS9 fibers and the softer SBR matrix. The SepS9 fibers are randomly oriented, without significant interaction with the rubber matrix. The lateral and longitudinal dimensions of the Sep rods are comparable with those obtained from the TEM images in Figure 6.

**Figure 8:**
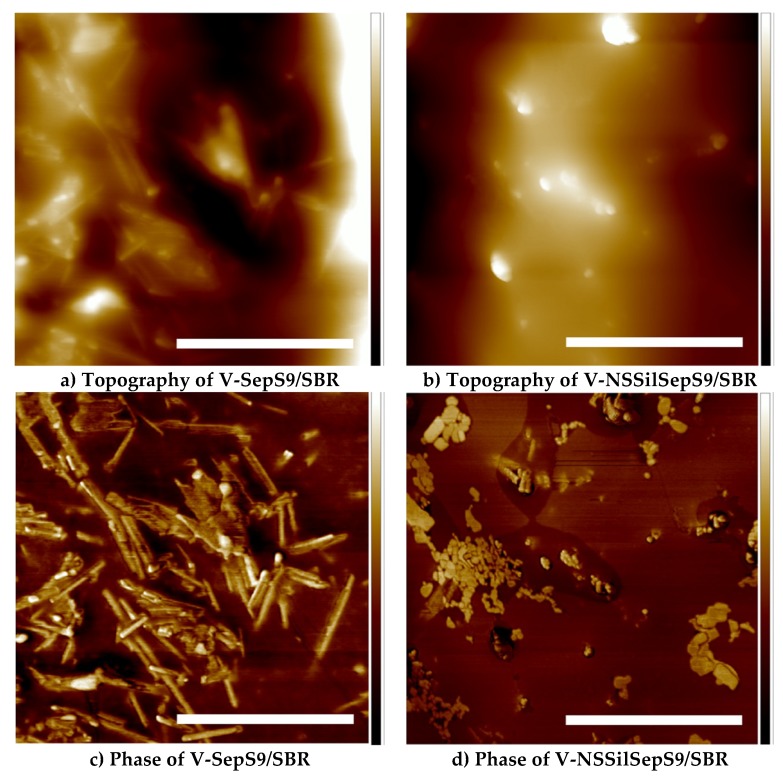
TM topographic and phase images of the cured composites: (**a**,**c**) V-SepS9/SBR and (**b**,**d**) V-NS-SilSepS9/SBR. The white scale bar is 1.0 µm. The Z-range is 100 nm and the phase scale is 25 degrees.

Figure 8 compares PNC prepared with pristine SepS9 with respect to that containing modified NS-SilSepS9 (AFM analyses of NS-SepS9 are reported in Figures S2 and S3 of SM). In line with the TEM images, significant differences in the AR between SepS9 and NS-SilSepS9 are detectable. The modified Sep nanorods in V-NS-SilSepS9/SBR appear embedded in a rigid zone as the particle boundaries seem less defined and are covered by rubber material linked to the Sep surfaces (Figure 8b,d). As recently reported in the literature [33], the self-assembly of fillers in a sheet or in a string-like structure give rise to NCs that have better mechanical properties despite a low dispersion. In fact, the larger amount of bounded rubber at the filler–rubber interface in the self-assembled filler structures enhances the amount of overlapping rubber layers and thus improves the mechanical properties.

**Figure 9:**
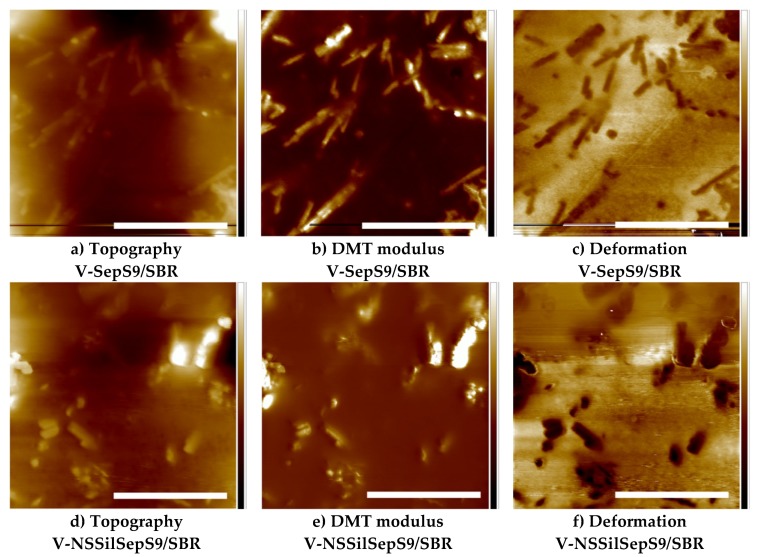
Peak force tapping images of V-SepS9/SBR and V-NS-SilSepS9/SBR. (**a**) and (**d**) topography, (**b**) and (**e**) Derjaguin, Muller, Toropov (DMT) modulus, and (**c**) and (**f**) deformation. Brighter colors indicate higher values of (**a**) surface height, (**b**) modulus, and (**c**) deformation. The white scale bar is 1.0 µm. The Z-range is 100 nm, the DMT range is 2.0 GPa, and the deformation range scale is 5 nm.

TM phase images are often very difficult to interpret since the phase signal is a convolution of several properties, such as the dissipation, stiffness, and adhesion. Thus, the PFT AFM technique was used to gain deeper insight into the nanomechanical properties of the samples. Figure 9 shows the topography, the rigid modulus, and the samples’ deformation as determined by fitting the force curve using the Derjaguin, Muller, Toropov (DMT) model, simultaneously [34].

From Figure 9a,d, by comparing the PNCs with unmodified SepS9 and modified NS-SilSepS9 fibers, one can clearly conclude that in the regions where the modulus and the deformation are the lowest, the average dimensions of Sep fibers are larger than those of the unmodified filler. This suggests the presence of a rubber “layer” of about 15 to 50 nm linked to the NS-SilSepS9 surface displaying more rigid properties than the SBR matrix. 

This rubber “layer” is constituted by heterogeneous rubber zones, surrounding the self-assembled Sep fibers, consisting of both the immobilized rubber chains, strongly interacting with the Sep filler through silane TESPT grafted on the surface, as detected by NMR, and the more loosely interacting outermost rubber chains, cross-linked or entangled with the primary layer.

The mapping of the deformation properties (Figure 9c,f) confirms these observations, indicating that the chemical modification of the Sep clay drastically affects the nanomechanical properties of the final PNC. 

To further refine the above results, ImAFM was performed in order to determine the nanomechanical properties based on (i) the measurement of the amplitude and phase images of the intermodulation products of two close frequencies, (ii) a proper calibration of the cantilever (for instance, by using the Sader’s method), and (iii) the determination of the detection sensitivity. 

**Figure 10:**
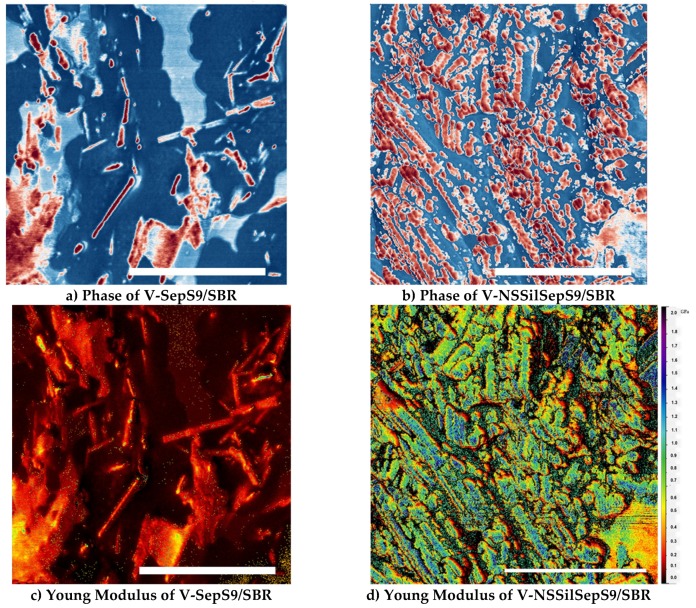
Intermodulation Atomic Force Microscopy (AFM) phase images of (**a**) V-SepS9/SBR, (**b**) V-NS-SilSepS9/SBR. Young modulus calculated from the DMT model and the intermodulation product amplitude and phase images of (**c**) V-SepS9/SBR, (**d**) V-NS-SilSepS9/SBR. The white scale bar is 1.0 µm. The phase scale is 25 degrees, and the Young modulus scale is 2.0 GPa.

Figure 10 shows the phase of the drive frequency of both V-SepS9/SBR and V-NS-SilSepS9/SBR PNCs. V-NS-SilSepS9/SBR (Figure 10b) shows the phase of the red area related to Sep particles larger than that of the blue area corresponding to the rubber, while in V-SepS9/SBR (Figure 10a), the blue areas have the largest phase. Moreover, as already observed from PFT AFM, for V-NS-SilSepS9/SBR, the red elongated zones are not well defined and the corresponding Sep rods present a rough surface at the filler–rubber interface due to the heterogeneous rubber zone surrounding the Sep nanoparticles.

One of the key aspects of ImAFM is the possibility to derive from the FI(A) and FQ(A) curves the nanomechanical properties and, in particular, the local Young modulus. Figure 10c,d represent the values calculated from the force curve for each pixel of the images, respectively, for V-SepS9/SBR and V-NS-SilSepS9/SBR PNCs. 

The Young modulus values are different between the zone of free rubber and the rubber close to the filler particles. In particular, V-NS-SilSepS9/SBR has an apparent Young modulus that gradually increases from the rubber matrix to the heterogeneous rubber zone around the filler and the more tight immobilized layer close to the modified Sep particles. In fact, the values change from 0.2 GPa (red in Figure 10d) to the highest value close to 1.8 GPa (blue). On the contrary, V-SepS9/SBR shows the lowest and more constant Young modulus close to the pristine Sep particles, around 0.2 GPa (red in Figure 10c). This confirms that modified Sep fibers supply remarkable fractions of immobilized rubber at the filler–rubber interface associated with the better mechanical properties, as described in the Section 3.1.

## 4. Conclusions

NS-SepS9/SBR PNCs were prepared by the blending method, using Sep clay anisotropic particles, obtained by applying a controlled surface acid treatment. The PNCs were used as model materials to investigate the influence of the clay distribution and dispersion on the clay/polymer interface and to study its effect on the dynamic-mechanical behavior of the rubber material. 

DMTA of both NS-SepS9/SBR and NS-SilSepS9/SBR samples highlights the improved balance between reinforcing and hysteretic properties of rubber materials, in comparison with the starting SepS9. 

Low-resolution ^1^H SS NMR revealed the presence of rigid rubber close to NS-SilSepS9 surfaces, associable to the increased chemical interaction between polymer and modified Sep fibers, having a high density of silanol groups on the surface with respect to the pristine Sep. Instead, for composites with untreated SepS9 fillers, the fraction of rigid rubber did not significantly vary with respect to pure SBR. Therefore, NMR supports that the modified Sep favors interfacial phenomena, in terms of filler/rubber interactions that affect the entire bulk, in line with the improvement of hysteretic behavior. It is worthy to note that until now, the reinforcement in rubber-clay PNCs has been mainly attributed to the geometric effect of stiff and well-dispersed filler particles. 

Morphological investigation showed that only the Sep fibers, able to interact with the rubber at the interface and that display reduced particle dimensions, succeeded in disrupting the tactoid interactions and favor self-assembly fibers in nanostructures inside the rubber matrix. This provides a larger amount of immobilized rubber at the filler–rubber interface, further enhancing the amount of overlapping rubber layers and thus improving the mechanical properties, as confirmed by AFM analysis.

The results of this integrated multi-technique approach indicate that the macroscopic mechanical properties of clay-based PNCs can be remarkably enhanced by self-assembled filler domains, whose formation and structure can be controlled by manipulating the chemistry at the hybrid interfaces between the filler particles and the polymers.

## Figures and Tables

**Table 1:** E′, E″, and Tan δ at 30 and 70 °C and Tg from E of SepS9 PNCs.

	E′ at 30 °C (MPa)	E′ at 70 °C (MPa)	E″ at 30 °C (MPa)	E″ at 70 °C (MPa)	Tan δ at 30 °C	Tan δ at 70 °C	Tg from E″ (°C)
**V-SBR-REF-0**	1.11	1.09	0.13	0.02	0.11	0.02	−10.73
**V-SepS9/SBR**	14.23	10.43	2.03	0.80	0.14	0.07	−7.1
**V-NS-SepS9/SBR**	4.99	3.89	0.62	0.25	0.12	0.06	−9.68
**V-NS-SilSepS9/SBR**	7.60	5.69	1.06	0.28	0.14	0.04	−7.44

**Table 2:** Molar percentages (%H) of ^1^H nuclei belonging to the filler (f_H_^Sep^) and of ^1^H nuclei in rigid environments, as determined by FID analysis using the procedure described in the text. f_Hrigid_^TOT^ is the total fraction of protons in rigid environments, while f_Hrigid_^Sep^ and f_Hrigid_^SBR^ are the fractions of filler and rubber protons in rigid environments, relative to the whole composite, respectively. f_Hrigid_^SBR*^ is the fraction of rubber protons in rigid environments relative to the sole SBR component.

	%H Relative to the Composite				%H Relative to SBR Only
Sample	f_Hrigid_^TOT^	f_H_^Sep^	f_Hrigid_^Sep^	f_Hrigid_^SBR^	f_Hrigid_^SBR*^
**SBR-REF-0**	6	-	-	6	6
**SepS9/SBR**	9	4	2	7	7
**NS-SepS9/SBR**	10	4	1	9	9
**NS-SilSepS9/SBR**	12	6	3	9	10
**V-SBR-REF-0**	12	-	-	12	12
**V-SepS9/SBR**	13	4	2	11	11
**V-NS-SepS9/SBR**	13	4	1	12	13
**V-NS-SilSepS9/SBR**	19	6	3	16	17

## Supplementary Materials

The following are available online at https://www.mdpi.com/2079-4991/9/4/486/s1, Table S1: Weight (w, %) and T_2_ values of the proton fractions, Table S2: Standard samples with known hydrogen content (H%, wt%), Figure S1: Calibration curve for the dependence of the total 1H FID intensity, Table S3: Results of the analysis of the ^1^H FID’s recorded for the pure Sep fillers, Figure S2: Tapping Mode topographic and phase images of cured composites V-SepS9/SBR, Figure S3: Intermodulation AFM phase images of V-NS-SepS9/SBR.

## References

[1] Di Credico B., Redaelli M., Bellardita M., Calamante M., Cepek C., Cobani E., D’Arienzo M., Evangelisti C., Marelli M., Moret M. (2018). Step-by-Step Growth of HKUST-1 on Functionalized TiO_2_ Surface: An Efficient Material for CO_2_ Capture and Solar Photoreduction. Catalysts.

[2] D’Arienzo M., Masneri V., Rovera D., Di Credico B., Callone E., Mascotto S., Pegoretti A., Ziarelli F., Scotti R. (2018). Tailoring the Dielectric and Mechanical Properties of Polybutadiene Nanocomposites by Using Designed Ladder-like Polysilsesquioxanes. ACS Appl. Nano Mater..

[3] D’Arienzo M., Diré S., Redaelli M., Borovin E., Callone E., Di Credico B., Morazzoni F., Pegoretti A., Scotti R. (2018). Unveiling the hybrid interface in polymer nanocomposites enclosing silsesquioxanes with tunable molecular structure: Spectroscopic, thermal and mechanical properties. J. Colloid Interface Sci..

[4] D’Arienzo M., Scotti R., Morazzoni F., Di Credico B., Giannini L. (2017). Silica-Polymer Interface and Mechanical Reinforcement in Rubber Nanocomposites. Hybrid Organic-Inorganic Interfaces.

[5] Di Credico B., Tagliaro I., Cobani E., Conzatti L., D’Arienzo M., Giannini L., Mascotto S., Scotti R., Stagnaro P., Tadiello L. (2018). A Green Approach for Preparing High-Loaded Sepiolite/Polymer Biocomposites. Nanomaterials.

[6] Roland C. (2015). Chapter 02163 - Reinforcement of Elastomers. Ref. Modul. Mater. Sci. Mater. Eng..

[7] Redaelli M., D’Arienzo M., Brus J., Di Credico B., Geppi M., Giannini L., Matejka L., Martini F., Panattoni F., Spirkova M. (2018). On the key role of SiO2@POSS hybrid filler in tailoring networking and interfaces in rubber nanocomposites. Polym. Test..

[8] Susanna A., D’Arienzo M., Di Credico B., Giannini L., Hanel T., Grandori R., Morazzoni F., Mostoni S., Santambrogio C., Scotti R. (2017). Catalytic effect of ZnO anchored silica nanoparticles on rubber vulcanization and cross-link formation. Eur. Polym. J..

[9] Scotti R., Conzatti L., D’Arienzo M., Di Credico B., Giannini L., Hanel T., Stagnaro P., Susanna A., Tadiello L., Morazzoni F. (2014). Shape controlled spherical (0D) and rod-like (1D) silica nanoparticles in silica/styrene butadiene rubber nanocomposites: Role of the particle morphology on the filler reinforcing effect. Polym. (United Kingdom).

[10] D’Arienzo M., Redaelli M., Callone E., Conzatti L., Di Credico B., Dirè S., Giannini L., Polizzi S., Schizzi I., Scotti R. (2017). Hybrid SiO2@POSS nanofiller: A promising reinforcing system for rubber nanocomposites. Mater. Chem. Front..

[11] Usuki A., Kojima Y., Kawasumi M., Okada A., Fukushima Y., Kurauchi T., Kamigaito O. (1993). Synthesis of nylon 6-clay hybrid. J. Mater. Res..

[12] Vaia R.A., Jandt K.D., Kramer E.J., Giannelis E.P. (1996). Microstructural Evolution of Melt Intercalated Polymer-Organically Modified Layered Silicates Nanocomposites. Chem. Mater..

[13] Lebaron P.C., Wang Z., Pinnavaia T.J. (1999). Polymer-layered silicate nanocomposites: An overview. Appl. Clay Sci..

[14] Chen B., Evans J.R.G., Greenwell H.C., Boulet P., Coveney P.V., Bowden A.A., Whiting A. (2008). A critical appraisal of polymer–clay nanocomposites. Chem. Soc. Rev..

[15] Vilgis T.A., Heinrich G., Klüppel M. (2009). Reinforcement of Polymer Nano-Composites: Theory, Experiments and Applications.

[16] Gandini A. (2015). The Surface and In-Depth Modification of Cellulose Fibers. Adv. Polym. Sci..

[17] Rooj S., Das A., Stöckelhuber K.W., Wang D.Y., Galiatsatos V., Heinrich G. (2013). Understanding the reinforcing behavior of expanded clay particles in natural rubber compounds. Soft Matter.

[18] Meyer J., Hentschke R., Hager J., Hojdis N.W., Karimi-Varzaneh H.A. (2017). A nano-mechanical instability as primary contribution to rolling resistance. Sci. Rep..

[19] Di Credico B., Cobani E., Callone E., Conzatti L., Cristofori D., D’Arienzo M., Dirè S., Giannini L., Hanel T., Scotti R. (2018). Size-controlled self-assembly of anisotropic sepiolite fibers in rubber nanocomposites. Appl. Clay Sci..

[20] Papon A., Saalwächter K., Schäler K., Guy L., Lequeux F., Montes H. (2011). Low-field NMR investigations of nanocomposites: Polymer dynamics and network effects. Macromolecules.

[21] Mujtaba A., Keller M., Ilisch S., Radusch H.J., Beiner M., Thurn-Albrecht T., Saalwächter K. (2014). Detection of surface-immobilized components and their role in viscoelastic reinforcement of rubber-silica nanocomposites. ACS Macro Lett..

[22] Borsacchi S., Sudhakaran U.P., Calucci L., Martini F., Carignani E., Messori M., Geppi M. (2018). Rubber-filler interactions in polyisoprene filled with in situ generated silica: A solid state NMR study. Polymers.

[23] Pittenger B., Erina N., Su C. (2012). Quantitative Mechanical Property Mapping at the Nanoscale with PeakForce QNM. Burker Appl. Note.

[24] Platz D., Thoĺn E.A., Pesen D., Haviland D.B. (2008). Intermodulation atomic force microscopy. Appl. Phys. Lett..

[25] Chartoff R.P., Menczel J.D., Dillman S.H. (2008). Dynamic Mechanical Analysis (DMA).

[26] Joseph P.V., Mathew G., Joseph K., Groeninckx G., Thomas S. (2003). Dynamic mechanical properties of short sisal fibre reinforced polypropylene composites. Compos. Part A Appl. Sci. Manuf..

[27] Mark J.E., Erman B., Roland M. (2013). The Science and Technology of Rubber.

[28] Martini F., Borsacchi S., Geppi M., Tonelli M., Ridi F., Calucci L. (2018). Monitoring the hydration of MgO-based cement and its mixtures with Portland cement by 1H NMR relaxometry. Microporous Mesoporous Mater..

[29] Hansen E.W., Kristiansen P.E., Pedersen B. (2002). Crystallinity of Polyethylene Derived from Solid-State Proton NMR Free Induction Decay. J. Phys. Chem. B.

[30] Martini F., Borsacchi S., Geppi M., Ruggeri G., Pucci A. (2014). Understanding the aggregation of bis(benzoxazolyl)stilbene in PLA/PBS blends: A combined spectrofluorimetric, calorimetric and solid state NMR approach. Polym. Chem..

[31] Tadiello L., D’Arienzo M., Di Credico B., Hanel T., Matejka L., Mauri M., Morazzoni F., Simonutti R., Spirkova M., Scotti R. (2015). The filler–rubber interface in styrene butadiene nanocomposites with anisotropic silica particles: morphology and dynamic properties. Soft Matter.

[32] Gusev A.A. (2006). Micromechanical Mechanism of Reinforcement and Losses in Filled Rubbers. Macromolecules.

[33] Li Y., Moll J., Schadler L.S., Liu H., Kumar S.K., Panagiotopoulos A.Z., Ilavsky J., Benicewicz B.C., Pryamitsyn V., Ganesan V. (2009). Anisotropic self-assembly of spherical polymer-grafted nanoparticles. Nat. Mater..

[34] Derjaguin B.V., Muller V.M., Toporov Y.P. (1975). Effect of contact deformations on the adhesion of particles. J. Colloid Interface Sci..

