# Supplementary material for: Hybrid Interface in Sepiolite Rubber Nanocomposites: Role of Self-Assembled Nanostructure in Controlling Dissipative Phenomena
Source: Nanomaterials (Basel). 2019 Mar 27;9(4):486. doi: 10.3390/nano9040486 (PMC6523625; doi:10.3390/nano9040486)
Supplement: Supplementary file 1 [file nanomaterials-09-00486-s001.pdf]

nanomaterials

Supplementary Materials

Hybrid Interface in Sepiolite Rubber Nanocomposites: Role of Self-Assembled Nanostructure in Controlling Dissipative Phenomena

Elkid Cobani [1], Irene Tagliaro [1], Marco Geppi [2], Luca Giannini [3], Philippe Leclère [4], Francesca Martini [2], Thai Cuong Nguyen [4], Roberto Lazzaroni [4], Roberto Scotti [1], Luciano Tadiello [3] and Barbara Di Credico [1,*]

[1] Department of Materials Science, INSTM, University of Milano-Bicocca, Via R. Cozzi, 55, 20125 Milano, Italy; e.cobani@campus.unimib.it (E.C.); i.tagliaro@campus.unimib.it (I.T.); roberto.scotti@unimib.it (R.S.)
[2] Dipartimento di Chimica e Chimica Industriale, University of Pisa, Via Moruzzi 13, 56124 Pisa, Italy.; marco.geppi@unipi.it (M.G.); francesca.martini@unipi.it (F.M.)
[3] Pirelli Tyre SpA, 20126 Milano, Italy; luca.giannini@pirelli.com (L.G.); luciano.tadiello@pirelli.com (L.T.)
[4] Service de Chimie des Matériaux Nouveaux, Centre d'Innovation et de Recherche en MAtériaux Polymères (CIRMAP), Université de Mons-UMONS, 7000 Mons, Belgium; Philippe.LECLERE@umons.ac.be (P.L.); cuong.nguyenthai@student.umons.ac.be (T.C.N.); roberto.lazzaroni@ umons.ac.be (R.L.)
* Correspondence: barbara.dicredico@unimib.it; Tel.: +39-02-64485189

1. SS NMR Hydrogen content of the sepiolite fillers from ^{1}H FID analysis

Table S1. Weight (*w*, %) and T_2 values (T_2, μs) of the proton fractions located in regions with different degree of mobility obtained for the investigated samples combining SE and CPMG experiments as described in the text. The sum of the weights of exp2 and exp3 components was taken as that of the longest exponential component determined by SE, while the ratios between exp2 and exp3 weights were obtained from CPMG curves.

<table>
<tr><th></th><th colspan="8">SE / CPMG results</th></tr>
<tr><th>Sample</th><th colspan="2">Gau</th><th colspan="2">exp1</th><th colspan="2">exp2</th><th colspan="2">exp3</th></tr>
<tr><td></td><td>w</td><td>T2</td><td>w</td><td>T2</td><td>w</td><td>T2</td><td>w</td><td>T2</td></tr>
<tr><td>SBR-REF-0</td><td>6</td><td rowspan="4">20</td><td>52</td><td rowspan="4">300</td><td>37</td><td rowspan="4">1200</td><td>5</td><td rowspan="4">6500</td></tr>
<tr><td>SepS9/SBR</td><td>9</td><td>48</td><td>39</td><td>4</td></tr>
<tr><td>NS-SepS9/SBR</td><td>10</td><td>49</td><td>36</td><td>5</td></tr>
<tr><td>NS-SilSepS9/SBR</td><td>12</td><td>48</td><td>35</td><td>5</td></tr>
<tr><td>V-SBR-REF-0</td><td>12</td><td rowspan="4">20</td><td>60</td><td>140</td><td>25</td><td>570</td><td>3</td><td>3400</td></tr>
<tr><td>V-SepS9/SBR</td><td>13</td><td>57</td><td rowspan="2">180</td><td>27</td><td rowspan="2">760</td><td>3</td><td rowspan="2">4500</td></tr>
<tr><td>V-NS-SepS9/SBR</td><td>13</td><td>58</td><td>26</td><td>3</td></tr>
<tr><td>V-NS-SilSepS9/SBR</td><td>19</td><td>57</td><td>130</td><td>22</td><td>580</td><td>2</td><td>3400</td></tr>
</table>

The total hydrogen content in each sepiolite filler was determined from the total intensity of the corresponding ^{1}H FID at ED = 0 (I_0) extrapolated with the procedure described in the main text. First, a calibration curve was built with the standard samples reported in Table S1, for which the weight and the total hydrogen content (H%, wt%) were known. A linear dependence of the measured I_0 to weight ratio on H% was found, as shown in Figure S1. The values of H% determined by interpolating the calibration curve for the different sepiolite fillers are reported in Table S2.

Table S2. Standard samples with known hydrogen content (H%, wt%): Adam. (Adamantane), HMB (hexamethylbenzene), IBU-S (Ibuprofen sodium salt), DSS (4,4-dimethyl-4-silapentane-1-sulfonic acid), and K_2HPO_4 (dipotassium hydrogenphosphate).

Standard	Purity (wt%)	H% (wt%)
Adam.	≥ 99	11.7
HMB	≥ 98.5	11.1
IBU-S	≥ 98	7.9
DSS	≥ 97	6.9
K_2HPO_4	≥ 98	0.6

In order to determine the fraction of protons in rigid environments, the ^{1}H FID's recorded for the pure Sep fillers were analysed performing a discrete fitting with a linear combination of analytical functions, as described in the main text. The results are reported in Table S2. For all the fillers the ^{1}H FID's could be well reproduced with a linear combination of one Gaussian function (Gau) with a short T_2 of ~ 18-20 μs, ascribable to dipolar coupled protons in rigid environments, and one or two exponential components with longer T_2's of ~ 60 (exp1) and 250-370 (exp2) μs, which can be ascribed to protons in more mobile environments and/or to isolated hydroxyl groups.

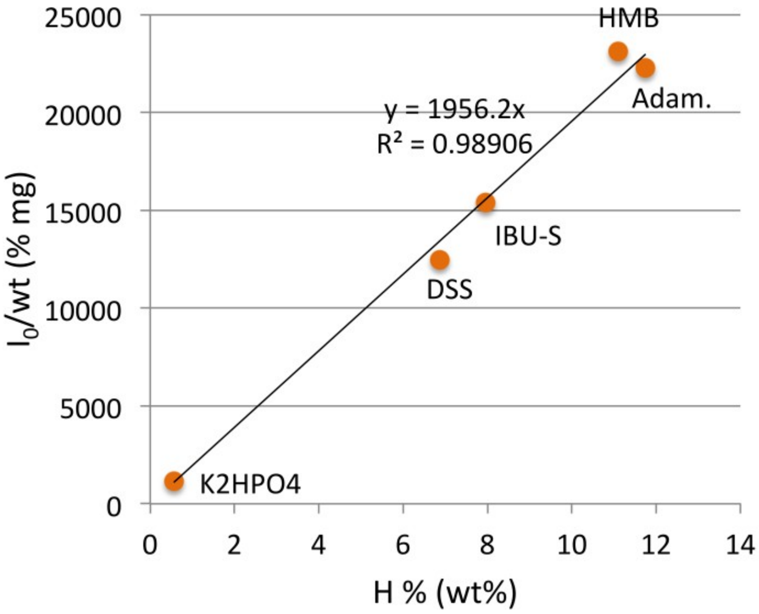

Figure S1. Calibration curve for the dependence of the total ^{1}H FID intensity to sample weight ratio (I_0/wt (%mg)) on the total hydrogen weight percentage (H%, wt%), built with the indicated standard samples.

Table S3. Results of the analysis of the ^{1}H FID's recorded for the pure Sep fillers. The total FID intensity to sample weght ratio (I_0/wt, %mg) and the total hydrogen content (H%, wt%) determined by interpolating the calibration curve in Table S1 are reported. For each component of the ^{1}H FID (Gau, exp1 and exp2) the values of weight (w) and spin-spin relaxation time (T_2) obtained by the fitting procedure are also shown.

Filler	Gau		exp1		exp2		I_0/wt	H% (wt%)
	w (%)	T_2 (μs)	w (%)	T_2 (μs)	w (%)	T_2 (μs)		
SepS9	48	20	52	60	-	-	3506	1.8
NS-SepS9	24	18	17	60	59	284	3358	1.7
NS-SilSepS9	62	18	17	60	22	249	4617	2.4

It is worth noticing that in order to calculate f_H^{Sep} in the composite samples the presence of TDAE extender oil in the amount of 37.5 phr had to be taken into account. Since the exact composition of TDAE, containing aromatic, naphtalenic and paraffinic components, is not known, an average hydrogen content of 10 % (H%, wt/wt), similar to that of SBR, was assumed for this oil. It was also verified that by varying H% in the range 9-11% the calculated values of the different proton fractions remain within the experimental error. Moreover, since TDAE was added in the same amount to all the samples, its contribution does not substantially affect the differences in terms of amount of rigid rubber observed among the samples]

2. AFM morphological analysis of V-NS-SepS9/SBR

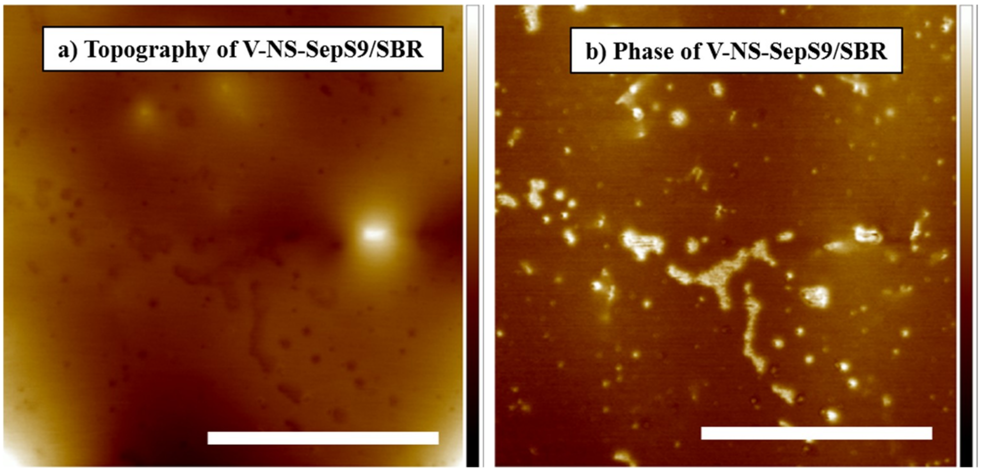

Figure S2. TM topographic (a) and phase images (b) of cured composites V-SepS9/SBR. The white scale bar is 1.0 μm. The Z-range is 100 nm and the phase scale is 25 degrees.

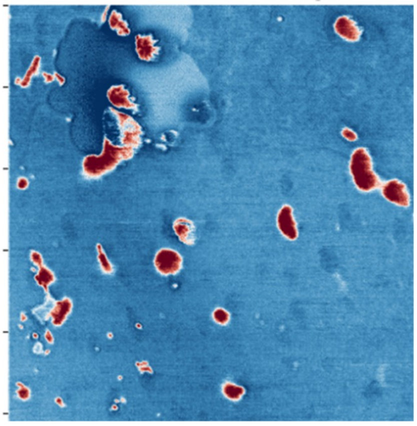

Figure S3. Intermodulation AFM phase images of V-NS-SepS9/SBR.
